# Chronic cyanosis since infancy: Unveiling a giant right pulmonary arteriovenous malformation

**DOI:** 10.1016/j.radcr.2025.06.084

**Published:** 2025-08-05

**Authors:** Dian Komala, Harry Galuh Nugraha, Firman Ramadhan, Rais Khairuddin, Rahmat Budi Kuswiyanto, Putria Rayani Apandi

**Affiliations:** a Department of Radiology, Faculty of Medicine, Universitas Padjadjaran, West Java, Indonesia; b Department of Pediatric, Faculty of Medicine, Universitas Padjadjaran, West Java, Indonesia

**Keywords:** Pulmonary arteriovenous malformation (PAVM), Chronic cyanosis, Computed tomography

## Abstract

Giant pulmonary arteriovenous malformations (PAVMs) are rare vascular anomalies involving direct connections between pulmonary arteries and veins, leading to right-to-left shunting and systemic hypoxemia. While small PAVMs may be asymptomatic, larger lesions often present with dyspnea, cyanosis, and related complications. An 18-year-old female with progressive dyspnea and cyanosis—first noted during infancy—was found to have a giant PAVM in the right lower lung lobe on thoracic computed tomography (CT). Imaging, particularly computed tomography (CT), was essential for diagnosis and treatment planning. This case underscores the need to consider PAVMs in the differential diagnosis of chronic cyanosis and highlights the critical role of radiologists in identifying and characterizing these lesions to guide appropriate management.and characterizing these lesions to support optimal clinical decision-making.

## Introduction

Pulmonary arteriovenous malformations (PAVMs) are abnormal vascular connections between pulmonary arteries and veins that bypass the normal capillary bed, resulting in a right-to-left shunt. While PAVMs vary in size, the term “giant” typically refers to lesions with an aneurysmal sac measuring 6 cm or more in diameter, although some experts use thresholds of 4-5 cm [1]. This distinction is clinically relevant, as giant PAVMs often present greater diagnostic and therapeutic challenges than smaller ones [1].

Due to their rarity, the epidemiology of giant PAVMs is not well-established, though case reports and small series suggest they represent less than 5% of all PAVMs [2]. With a general incidence of 2-3 per 100,000 population, giant PAVMs likely affect only a few individuals per million [3]. These lesions show a slight female predominance, with a female-to-male ratio of approximately 1.5-2:1 [4], and are most commonly diagnosed in adults aged 30-50, though cases have been reported across all age groups [5].

Giant PAVMs result from congenital vascular abnormalities that create low-resistance channels, which enlarge over time due to hemodynamic stress [6]. Their large size exacerbates the right-to-left shunt, intensifying symptoms such as cyanosis, dyspnea, hemoptysis, orthodeoxia, and exercise intolerance [7]. Chronic hypoxemia can lead to polycythemia and increased thrombotic risk, while paradoxical embolization can result in neurological complications, including stroke, seizures, and migraines [8]. Cardiovascular manifestations may include palpitations and a hyperdynamic precordium, and spontaneous rupture may cause life-threatening hemoptysis or hemothorax [9].

Although contrast-enhanced chest CT remains the gold standard for PAVM diagnosis, other imaging modalities are also useful. Transthoracic contrast echocardiography (TTCE) is a sensitive screening tool for right-to-left shunts and can help grade shunt severity. Time-resolved 3D magnetic resonance angiography (MRA) provides a radiation-free alternative for assessing PAVM patency and planning embolization. Digital subtraction pulmonary angiography, though less common for initial diagnosis, remains essential for assessing flow dynamics and guiding endovascular treatment. Dual-energy CT can help differentiate PAVMs from mimics such as calcified granulomas [7].

This report presents a case of a giant PAVM in a young female, emphasizing the importance of considering PAVMs in patients with longstanding cyanosis and dyspnea. Notably, initial echocardiography revealed normal cardiac structure and function, underlining the importance of further imaging in cases of unexplained cyanosis. The primary aim is to provide a comprehensive clinical and radiological overview of a giant PAVM, highlighting diagnostic approaches and imaging findings.

## Case report

### Clinical presentation

An 18-year-old female presented with progressive dyspnea and cyanosis, which had worsened over the previous month. Cyanosis had been noted during infancy, particularly at 5 months of age, manifesting around the mouth, hands, and feet during crying episodes. There was no history of fever or systemic symptoms, although mild epigastric discomfort and fatigue were reported. Physical examination revealed cyanosis of the lips, hands, and feet. Patient was lost to follow-up and could not be further evaluated.

### Diagnostic evaluation

**Fig. 1:**
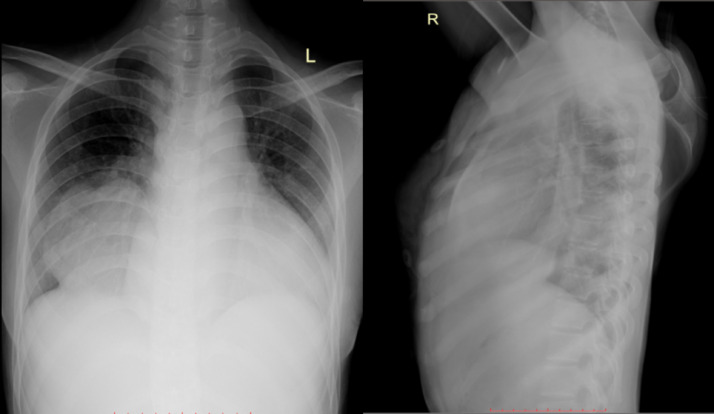
PA chest X-ray shows a mass obscuring the right hemidiaphragm, while the lateral view demonstrates a mass in retrocardiac area.

**Fig. 2:**
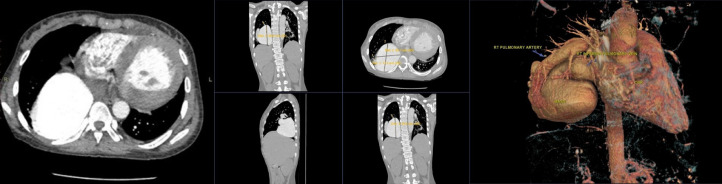
CT of PAVM.

Echocardiography revealed normal cardiac anatomy and function. A plain chest radiograph showed a well-defined, lobulated opacity with an obtuse angle relative to the mediastinum in the middle to lower zone of the right lung, suggestive of a mass. Cardiac border is visible on the posteroanterior view. This suggests the lesion is likely located in the right lower lobe or middle mediastinum (Fig. 1). CT demonstrated an enlarged right peripheral pulmonary artery with early filling of the right inferior pulmonary vein, consistent with a PAVM. Diameter of the lesion is 8.6 cm, with segmental branch of the lower lobe pulmonary artery as the feeding vessel (Fig. 2).

## Discussion

The clinical features in this case—chronic cyanosis since infancy and progressively worsening dyspnea—are consistent with a giant PAVM. These symptoms result from right-to-left shunting, which bypasses the pulmonary capillary bed and reduces arterial oxygenation. The absence of fever or systemic complaints aligns with the localized nature of PAVMs. The patient’s symptoms likely worsened due to gradual lesion enlargement, possibly influenced by hormonal changes. Mild epigastric discomfort and fatigue may have resulted from chronic hypoxemia [1].

This case illustrates the diagnostic complexity of PAVMs, particularly when cyanosis presents early in life. Normal echocardiographic findings prompted further imaging, leading to the identification of a right lung mass. Although chest radiography raised suspicion, it lacked specificity. Pulmonary CT angiography was crucial in confirming the diagnosis by identifying hallmark features: an enlarged pulmonary artery and early venous filling [10].

The case emphasizes a systematic diagnostic approach for chronic cyanosis, especially in infancy. While echocardiography and chest radiographs are useful initial tools, CT angiography remains the gold standard for diagnosis, anatomical characterization, and treatment planning. Given the broad differential diagnosis of right lung masses—including tumors, infections, and vascular anomalies—advanced imaging is essential [11]. In this case, the X-ray still suggested a broad differential diagnosis, which was confirmed by CT.

The pathophysiology of PAVMs involves direct communication between pulmonary arteries and veins, causing a right-to-left shunt that reduces systemic oxygenation and leads to cyanosis. Symptom severity correlates with lesion size and number. While PAVMs may initially reduce pulmonary arterial pressure by offering a low-resistance pathway, chronic shunting may eventually cause pulmonary hypertension, as seen in this case. Complications include paradoxical embolism, stroke, brain abscess, high-output heart failure, hemoptysis, and hemothorax. Chronic hypoxemia may also lead to secondary erythrocytosis and increased thrombotic risk, highlighting the need for timely diagnosis and treatment [1].

Quality of life is significantly affected in patients with PAVMs, as seen here with lifelong cyanosis and limited physical endurance. Chronic hypoxemia reduces exercise capacity, potentially impacting cognitive function and daily activities. Long-standing PAVMs may induce compensatory polycythemia, further increasing thrombotic risk [12]. Management requires a multidisciplinary approach, including regular oxygen saturation monitoring, exercise tolerance assessments, and neurologic screening. Treatment options include transcatheter embolization to occlude the shunt and improve oxygenation, along with supportive therapies such as supplemental oxygen, pulmonary rehabilitation, and psychological support. Patient education and prophylactic antibiotics for invasive procedures are also critical [13].

Radiologists are pivotal in diagnosing and managing PAVMs, particularly giant ones. Their ability to characterize lesion size, feeding arteries, and draining veins using CT angiography directly informs treatment strategies, especially when endovascular embolization is considered. They also help identify associated vascular anomalies that may affect management. By delivering detailed imaging assessments, radiologists support clinicians in tailoring individualized treatment plans [14,15].

The study by Lin et al. provides an example of term neonate presented with severe cyanosis. Initial evaluation with chest radiography revealed cardiomegaly, left lower lobe opacification, and rightward tracheal deviation—nonspecific but concerning findings. Despite normal intracardiac anatomy, key findings included a markedly dilated left atrium, enlarged left pulmonary veins, and evidence of torrential pulmonary venous return crossing the patent foramen ovale, indicating a left atrial volume overload. Angiography confirmed a large, multilobar PAVM in the left lower lobe with a feeder vessel measuring 9.5 mm in diameter and 2 draining lobes. Radiologic evaluation was central to the diagnosis and management of this life-threatening condition. Echocardiography enabled rapid bedside diagnosis and triage, while angiography provided both definitive imaging and a therapeutic route [16].

## Conclusion

This case of a giant PAVM underscores the critical role of advanced imaging in diagnosis and management. Radiologists play a central role in identifying lesion size, vascular anatomy, and associated anomalies, guiding appropriate interventions such as embolization or surgery. Lifelong follow-up is essential to monitor for recurrence or new malformations, ensuring optimal patient outcomes.

## Patient consent

Written informed consent for the publication of this case report was obtained from the patient

## References

[1] Salibe-Filho W., Oliveira F.R. (2023). Terra-Filho M. Update on pulmonary arteriovenous malformations. J Bras Pneumol.

[2] van Dantzig P., Quincey V., Kurz J., Ming C., Kamalaksha S., White D. (2024). Epidemiology of giant cell arteritis in Waikato, Aotearoa New Zealand. N Z Med J.

[3] Bodilsen J., Madsen T., Brand C.T., Petersen M.B., Hansen L.K., Nielsen J. (2024). Pulmonary arteriovenous malformations in patients with previous brain abscess: a cross-sectional population study. Eur J Neurol.

[4] Weng S.S., Cao Y., Tang X.J., Li X.Y., Zhang Q., Liu Z.H. (2017). Epidemiological features of pulmonary giant cell carcinoma: therapeutic patients with EGFR mutation-based case reports from the Surveillance, Epidemiology, and End Results database. Oncotarget.

[5] Aram S., Bhyravavajhala S., Vanaparty B., Narayanan R., Yerram S. (2022). Severe pulmonary arterial hypertension in a patient with hereditary hemorrhagic telangiectasia: multiple pulmonary and hepatic arteriovenous malformations. Ann Pediatr Cardiol.

[6] Spearman A.D., Gind S. (2022). Pulmonary vascular sequelae in palliated single-ventricle circulation: aortopulmonary collaterals and arteriovenous malformations. J Cardiovasc Dev Dis.

[7] Van den Heuvel D.A.F., Post M.C., Koot W., Janssen M., Steggerda J.A., Timmermans J. (2020). Comparison of contrast-enhanced MR angiography to CT for detecting pulmonary arteriovenous malformations. J Clin Med.

[8] Tagliapietra M., Turri G., Bortolotti F., Mansueto G., Monaco S. (2022). Ischemic stroke due to sporadic genetic pulmonary arteriovenous malformations: case report. Brain Circ.

[9] Ma M.R., Shi L., Wang F., Cai X., Ma L. (2024). Complex pulmonary artery fistula presenting with ventricular tachycardia type 2 acute myocardial infarction. Cureus.

[10] Pejhan S., Rahmanijoo N., Farzanegan R., Rahimi M. (2012). Surgically treatable pulmonary artery fistula. Ann Thorac Cardiovasc Surg.

[11] Müller-Hülsbeck S., Marques L., Maleux G., Cohen M.C., Preissel P., Noeldge G. (2020). CIRSE standards of practice on diagnosis and treatment of pulmonary arteriovenous malformations. Cardiovasc Intervent Radiol.

[12] Yu Q., Zangan S., Funaki B. (2024). Preliminary experience with a low-profile high-density braid occluder for transcatheter embolization of pulmonary arteriovenous malformations. J Vasc Interv Radiol.

[13] Kramdhari H., Valakkada J., Ayyappan A. (2021). Diagnosis and endovascular management of pulmonary arteriovenous malformations. Br J Radiol.

[14] Hong J., Lee S.Y., Lim J.K., Park H.J., Kim Y.J., Choi S.H. (2022). Feasibility of single-shot whole-thorax time-resolved MR angiography to evaluate patients with multiple pulmonary arteriovenous malformations. Korean J Radiol.

[15] Shin S.M., Kim H.K., Crotty E.J., Hammill A.M., Wusik K., Kim DH. (2020). CT angiographic findings of pulmonary arteriovenous malformations in children and young adults with hereditary hemorrhagic telangiectasia. AJR Am J Roentgenol.

[16] Lin Y., Hogan W., Stillwell K., Moore P., Peyvandi S., Amin E., Quezada E. (2020). Giant neonatal pulmonary arteriovenous malformation: an imaging and management challenge. CASE (Phila).

